# Simultaneous Fifth Carpometacarpal Joint Dislocation and Fourth Metacarpal Neck Fracture: A Conservative Treated Case With a Functional Metacarpal Splint

**DOI:** 10.7759/cureus.45566

**Published:** 2023-09-19

**Authors:** Muhammed Yusuf Afacan, Abdısalam Mutaj Shafaj Nur, Tural Vahitoglu

**Affiliations:** 1 Department of Orthopedics and Traumatology, Istanbul University Cerrahpasa, Cerrahpasa Medical Faculty, Istanbul, TUR; 2 Department of Orthopedics and Traumatology, Silvan Dr. Yusuf Azizoglu State Hospital, Diyarbakır, TUR

**Keywords:** muscle strengthening exercises, range of motion exercises, conservative treatment, longitudinal traction, closed fracture reduction, functional metacarpal splint, metacarpal neck fracture, carpometacarpal joint dislocation

## Abstract

Simultaneous carpometacarpal joint dislocation and fractures of adjacent carpal bones are rare orthopedic injuries. With this case, we aimed to discuss the effectiveness and ergonomics of the functional metacarpal splint in carpometacarpal joint dislocations and metacarpal neck fractures without surgery. A 27-year-old right-hand dominant male applied to the emergency department after a punch on a wall with his right fist. Pain, swelling, and deformity were evident without neurovascular injury. The radiographs showed simultaneous fifth carpometacarpal joint dislocation and fracture of the neck of the fourth metacarpal bone. We performed closed reduction with longitudinal traction and applied a functional metacarpal splint. We followed up with the patient regularly, and on the fourth week, we removed the splint and began a range of motion exercises. On the sixth week, we began muscle strengthening exercises, and we reached full range of motion with fair muscle strength on the eighth week of the follow-up without any deformity. In this case, prompt diagnosis, longitudinal traction, closed reduction with manual dorsal manipulation, and functional metacarpal splinting were adequate. We achieved a full range of motion without the need to immobilize the wrist or metacarpophalangeal joints or undergo surgery after proper immobilization with a functional metacarpal splint.

## Introduction

Simultaneous carpometacarpal joint dislocation and adjacent metacarpal neck fracture is a rare injury pattern, which we may miss due to edema in the hand and inadequate X-ray interpretation [1]. Delayed diagnosis is associated with high morbidity due to posttraumatic arthritis, which results in pain and reduced grip strength [2]. The damage mechanism is typically a combination of axial loading and rotational forces on the hand. While these damages usually occur because of high-energy trauma, they may also occur because of the patient punching the wall. Depending on the force's direction, these dislocations are divided into dorsal and volar dislocations with or without fracture [3]. Compared to the volar dislocation, the dorsal dislocation occurs more frequently [4]. A high index of clinical suspicion with a thorough physical examination and proper radiological evaluation can help to diagnose. A plain X-ray should include anteroposterior, oblique, and lateral views with a systematic assessment to look for joint incongruence. By presenting this case, we wanted to show whether closed reduction and functional metacarpal splint application were sufficient as treatment in a case with adjacent metacarpal neck fracture accompanying carpometacarpal joint dislocation and whether the patient could regain his previous joint range of motion and muscle strength without the need for surgical treatment. In addition, we wanted to bring this rare fracture-dislocation case and the success achieved with conservative treatment to the literature.

## Case presentation

**Figure 1:**
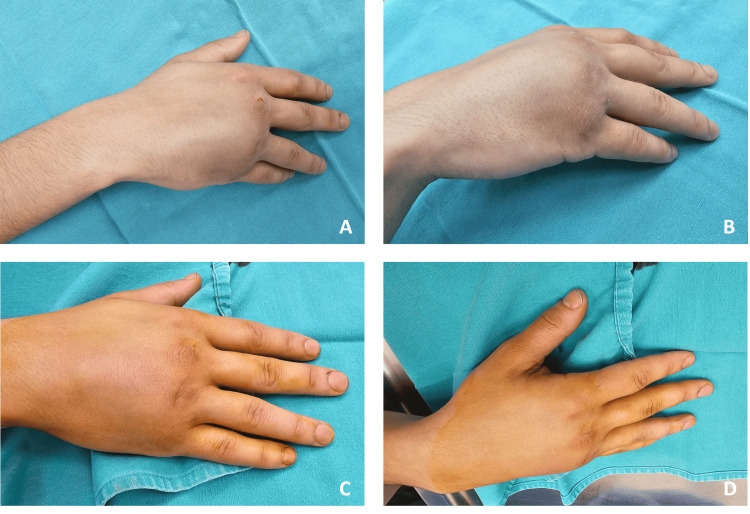
Hand photographs of the patient before reduction show swelling and evident deformity Figure 1A: Anterior hand photograph of the patient before reduction. Figure 1B: Lateral hand photograph of the patient before reduction. Figure 1C: Anterior hand photograph of the patient after reduction. Figure 1D: Lateral hand photograph of the patient after reduction.

A 27-year-old, right-hand dominant male applied to the emergency department with severe pain, swelling, and deformity of his right hand (Figure 1).

**Figure 2:**
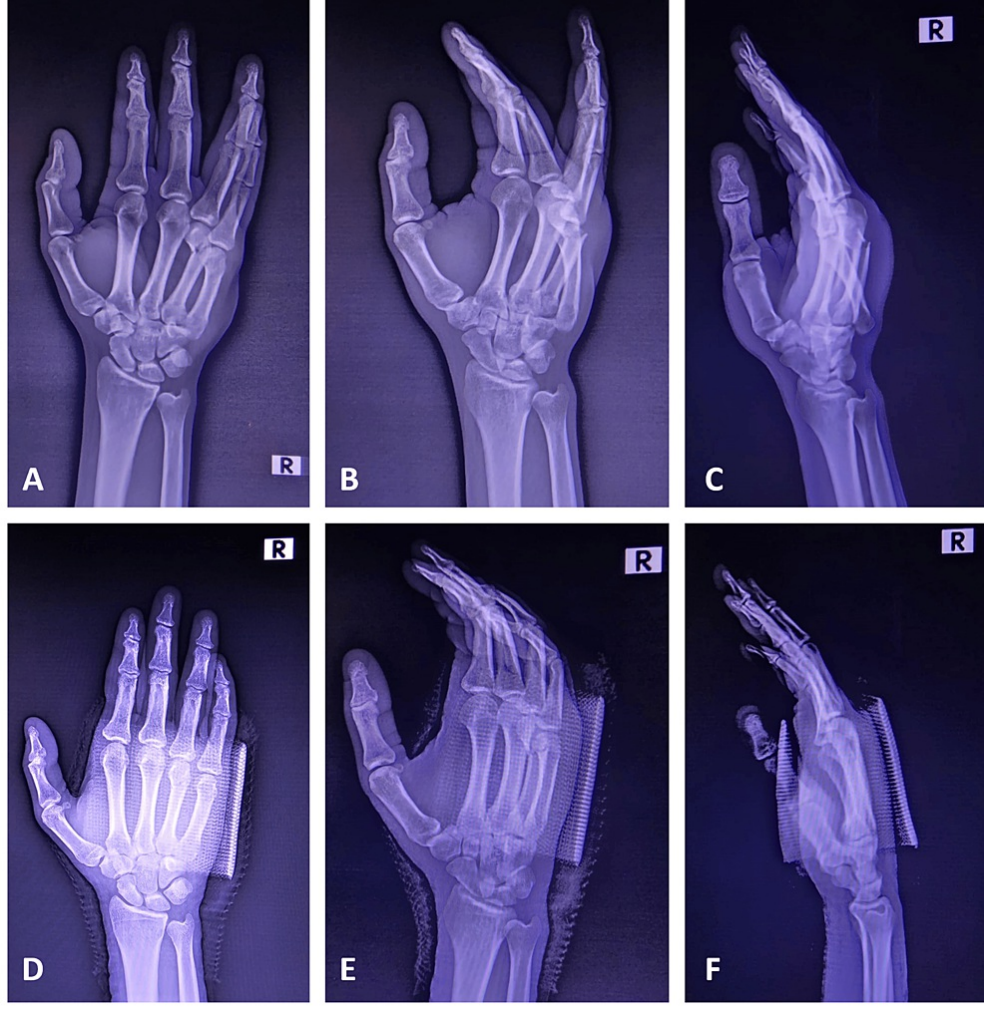
Hand radiographs of the patient before and after reduction Figure 2A: Hand anteroposterior view of the patient before reduction, showing simultaneous fifth carpometacarpal joint dislocation and fourth metacarpal neck fracture. Figure 2B: Hand oblique view of the patient before reduction, showing simultaneous fifth carpometacarpal joint dislocation and fourth metacarpal neck fracture. Figure 2C: Hand lateral view of the patient before reduction, showing simultaneous fifth carpometacarpal joint dislocation and fourth metacarpal neck fracture. Figure 2D: Hand anteroposterior view of the patient with a functional metacarpal splint after reduction, showing proper alignment of the carpal and metacarpal bones. Figure 2E: Hand oblique view of the patient with a functional metacarpal splint after reduction, showing proper alignment of the carpal and metacarpal bones. Figure 2F: Hand lateral view of the patient with a functional metacarpal splint after reduction, showing proper alignment of the carpal and metacarpal bones.

It happened following a punch on a wall with a clenched fist. The physical examination revealed a painful, slightly swollen hand with an evident deformity at the ulna-dorsal aspect of his hand. The little finger and the fifth metacarpal were deviated ulnarly and dorsally. There was no neurologic or vascular deficit. The anteroposterior, oblique, and lateral hand radiographs revealed dorsal fifth carpometacarpal joint dislocation with an associated fourth metacarpal neck fracture (Figure 2).

**Figure 3:**
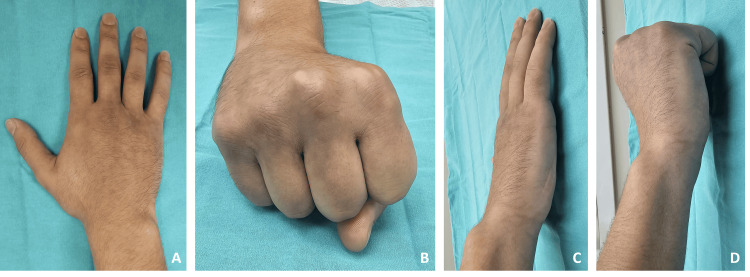
Hand photographs of the patient at the end of the sixth week Figure 3A: Anterior photograph of the patient's hand at the end of the sixth week exhibiting a normal appearance. Figure 3B: Anterior photograph of the patient's fist at the end of the sixth week exhibiting a normal appearance. Figure 3C: Lateral photograph of the patient's hand at the end of the sixth week exhibiting a normal appearance. Figure 3D: Lateral photograph of the patient's fist at the end of the sixth week exhibiting a normal appearance.

After the closed reduction with manipulation at the emergency department, a functional metacarpal splint was applied to the patient to stabilize the carpometacarpal joint and immobilize the fourth metacarpal neck. Simultaneous longitudinal traction of the fourth and the fifth fingers with dorsal pressure on the fifth metacarpal base enabled close reduction. After the closed reduction, the control plain X-ray showed an anatomic reduction with normal joint anatomy and proper alignment without neurologic deficit. We recommended the patient ice application, elevation, neurovascular follow-up, appropriate analgesia with paracetamol if required, and follow-up at the orthopedics and traumatology outpatient clinic. A conservative management with the functional metacarpal splint was sufficient, and we followed up with the patient weekly at the outpatient clinic. On the fourth week, we removed the metacarpal splint, and the patient was started on physical rehabilitation with a range of motion exercises because the callus formation was sufficient on the anteroposterior, oblique, and lateral radiographs, and the bony union had occurred. In the sixth week, the hand had a normal appearance (Figure 3).

**Figure 4:**
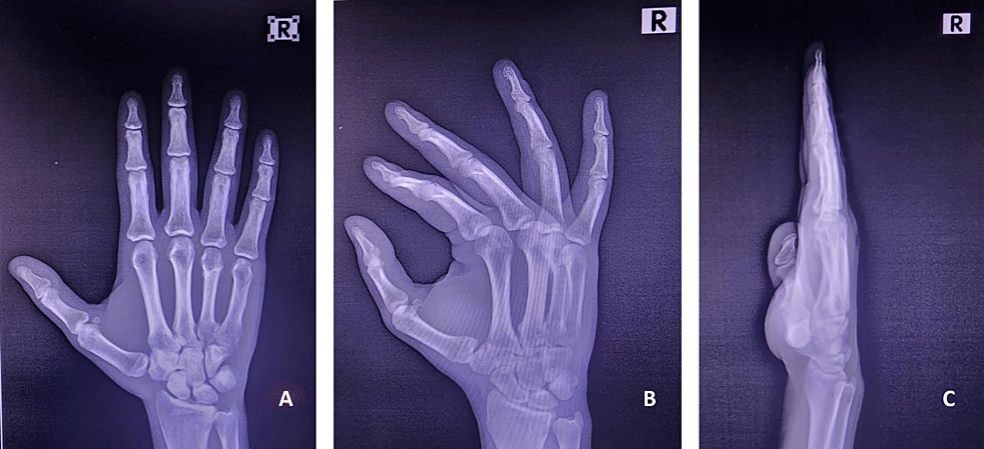
Hand radiographs of the patient at the end of the sixth week Figure 4A: Hand anteroposterior view of the patient at the end of the sixth week showing the union of the fourth metacarpal neck and proper alignment of the carpal and metacarpal bones. Figure 4B: Hand oblique view of the patient at the end of the sixth week showing the union of the fourth metacarpal neck and proper alignment of the carpal and metacarpal bones. Figure 4C: Hand lateral view of the patient at the end of the sixth week showing the union of the fourth metacarpal neck and proper alignment of the carpal and metacarpal bones.

The radiographs also showed a bony union with the proper alignment (Figure 4).

We also started strengthening exercises in the sixth week. We achieved a full range of motion with normal grip strength on the eighth week of follow-up without residual deformity.

## Discussion

Carpometacarpal joint injuries are rare, representing less than 1% of all hand injuries [5]. They occur typically due to high-energy trauma like road traffic accidents or falls from heights, where significant force is required to disrupt the strong, peri-articular ligamentous support [6]. However, lower energy mechanisms, such as punching in a clenched fist, can also lead to this condition. The patient hit the wall with a clenched fist, resulting in the simultaneous fifth carpometacarpal joint dislocation and fourth metacarpal neck fracture in our case. The causative injury can involve direct force applied at the metacarpal bases or indirectly through the metacarpal shaft. Depending on the applied force and the wrist position during the injury, injuries to the hand can result in either volar or dorsal carpometacarpal dislocation [4]. The carpometacarpal joint stability is provided by volar and dorsal ligaments, by interlocking saddle joints, intermetacarpal ligaments, the long flexor and extensor tendons, and intrinsic hand muscles [2]. Two factors create instability at the carpometacarpal joint of the fifth finger. One is its resemblance to the thumb carpometacarpal joint, which is a saddle-type joint, and gives it greater mobility compared to the central three carpometacarpal joints. The other one is the insertion of the flexor carpi ulnaris tendon at the base of the fifth carpometacarpal joint and the fact that the fifth metacarpal configuration slopes toward the ulnar side, whereas the fourth carpometacarpal joint is transverse [5]. Therefore, the ulnar slope of the fifth metacarpal and the insertion of the flexor carpi ulnaris tendon play a role in creating instability in the fifth carpometacarpal joint compared to the central three carpometacarpal joints and, hence, predisposes it to dislocation [5,7]. A recent prospective comparative study that compared the use of the functional metacarpal splint and ulnar gutter splint in the treatment of the fifth metacarpal neck fractures showed long-term similar clinical and radiologic outcomes with the functional metacarpal splint, which yielded early improvement in clinical scores and hand grip strength [8]. In the current case, the fourth metacarpal neck fracture was also present. Therefore, we preferred the functional metacarpal splint to immobilize the metacarpals and the carpometacarpal joint. The patient exhibited excellent radiologic and clinical outcomes without the need for surgery.

## Conclusions

Simultaneous carpometacarpal joint dislocation and accompanying adjacent metacarpal neck fracture is a rare orthopedic emergency that needs immediate intervention. Prompt diagnosis, longitudinal traction, and closed reduction with manual dorsal manipulation followed by a functional metacarpal splint were sufficient as treatment. The patient was followed up with a functional metacarpal splint covering the hand, and a full range of motion was achieved without the need to immobilize the wrist or metacarpophalangeal joints and without the need for surgical intervention.
